# FiO_2_ requirements during general anesthesia with dual-lung ventilation: a prospective pilot study

**DOI:** 10.3389/fmed.2026.1780890

**Published:** 2026-03-09

**Authors:** Jie-feng Sun, Jia-bao Chen, Wei-long Wang, Hong-fa Wang, Jin-tao Liu, Mei Cheng, Zhen-feng Zhou

**Affiliations:** 1 Department of Anesthesiology, Hangzhou Fuyang Hospital of Traditional Chinese Medicine, Hangzhou, China; 2 The Fourth Clinical Medical College, Zhejiang Chinese Medicine University (Hangzhou First People’s Hospital), Hangzhou, Zhejiang, China; 3 Center for Rehabilitation Medicine, Department of Anesthesiology, Zhejiang Provincial People’s Hospital (Affiliated People’s Hospital, Hangzhou Medical College), Hangzhou, China; 4 Department of Anesthesiology, Hangzhou Women’s Hospital (Hangzhou Maternity and Child Health Care Hospital, Hangzhou First People’s Hospital Qianjiang New City Campus, Zhejiang Chinese Medical University), Hangzhou, China

**Keywords:** general anesthesia, inspiratory oxygen fraction, oxygen exposure, oxygen saturation, partial pressure of oxygen, ventilation

## Abstract

**Background:**

Hyperoxemia and prolonged oxygen exposure were common during general anesthesia. However, the relationship between FiO_2_ and PaO_2_ in patients undergoing general anesthesia with dual lung ventilation remained unclear. This prospective pilot study aimed to explore this relationship.

**Methods:**

A cohort of 50 patients was recruited for this self-controlled, prospective pilot study. A standardized volume-controlled ventilation strategy was applied, with FiO_2_ initially set to 0.3 immediately after tracheal intubation. FiO_2_ was then increased in steps of 0.1 until it reached 0.6, followed by an increase to 0.8. Each FiO_2_ step was maintained for at least 30 min before blood samples were drawn for blood gas analysis at each point.

**Results:**

Rmcorr analyses revealed a significant correlation between FiO_2_ and PaO_2_ (*p* < 0.001). The correlation coefficient (*r*) was 0.967 and Model convergence was robust, with a gradient value of 0.00003. At an FiO_2_ of 40, 78.0% of patients maintained PaO_2_ between 100 and 200 mmHg, while more than 68.0% exceeded 200 mmHg at 50% FiO_2_ levels. Rmcorr analysis revealed a weak but statistically significant correlation between FiO_2_ and PaO_2_/FiO_2_ (*r* = 0.290; *p* < 0.001).

**Conclusion:**

A significant linear correlation was identified between FiO_2_ and PaO_2_ during general anesthesia with dual-lung ventilation. In this prospective pilot study, our findings suggested that maintaining 40% FiO_2_ was generally sufficient for most patients to achieve PaO_2_ levels of 100–200 mmHg, unless specific clinical conditions require otherwise. Importantly, FiO_2_ need not exceed 50% in most patients undergoing general anesthesia with dual-lung ventilation.

**Clinical trial registration:**

http://www.chictr.org.cn, identifier ChiCTR2500095383.

## Introduction

Pulmonary atelectasis occurred in over 90% of patients undergoing general anesthesia, with high inspiratory oxygen fraction (FiO_2_) identified as a major contributing factor (1). Recent randomized clinical trial (2) and retrospective study (1) have highlighted the association between elevated intraoperative FiO_2_ and an increased risk of major respiratory complications and mortality, often in a dose-dependent manner. These findings suggested that intraoperative FiO_2_ should be carefully adjusted to just above the level required to maintain adequate arterial oxygen saturation (2).

The British Thoracic Society guidelines (3) recommend titrating FiO_2_ to achieve normal PaO_2_ levels, as supraphysiological oxygen concentrations were commonly observed in patients unless a clear clinical benefit from high FiO_2_ was evident. Further studies suggested that intraoperative PaO_2_ should be maintained within the range of 100–200 mmHg (4). However, oxygen saturation (SpO_2_) monitoring, the most commonly used method in clinical practice, provided limited insight into arterial blood oxygen levels (PaO_2_), particularly when SpO_2_ was ≥98% during general anesthesia (4). Additionally, arterial blood gas (ABG) analysis, though informative, was invasive and not routinely required for all patients.

Several theoretical models (5–10) have been proposed to predict the relationship between FiO_2_ and PaO_2_. However, most of these models have not been widely accepted in clinical practice and are often tailored to ICU patients receiving various modes of ventilatory support.

A multicenter, cross-sectional observational study (11) also found that hyperoxemia and excessive oxygen exposure are common during general anesthesia. Despite this, the precise relationship between FiO_2_ and PaO_2_ in patients undergoing general anesthesia with dual-lung ventilation remained unclear. Therefore, we conducted this prospective pilot study to further investigate this relationship.

## Methods

### Study design

This prospective pilot study was approved by the Ethics Committee of Zhejiang Provincial People’s Hospital and conducted between January 07 and February 18, 2025 at Zhejiang Provincial People’s Hospital, China.

Eligible patients were aged 18 years or older, with an American Society of Anesthesiologists (ASA) physical status of I-III, scheduled for elective surgery under general anesthesia expected to last longer than 3 h. All patients required radial artery puncture, central venous catheterization, and had preoperative PaO_2_ > 60 mmHg.

Exclusion criteria included emergency surgery, prior lung surgery, need for non-invasive oxygen therapy, chronic obstructive pulmonary disease (COPD), acute respiratory failure (including acute lung injury, pneumonia, or acute respiratory distress syndrome), severe cardiac disease or persistent hemodynamic instability, requirement for renal replacement therapy (CRRT), sepsis or septic shock, progressive neuromuscular disorders, pregnancy, participation in another clinical trial, or refusal to participate.

### Anesthesia and monitoring

Prior to anesthesia induction, an internal jugular vein catheter and radial artery catheter were inserted. Standard anesthesia monitoring included SpO_2_, ECG, arterial blood pressure, heart rate (HR), end-tidal carbon dioxide (EtCO_2_), and bispectral index (BIS), all recorded using a Datex Ohmeda S/5 Avance monitor (GE Healthcare, Helsinki, Finland).

Crystalloid solution (12–15 mL/kg/h) and vasoactive drugs were infused to maintain the mean arterial pressure (MAP) at 80%–90% of the patient’s baseline blood pressure, defined as the blood pressure measured in a calm state before surgery. Blood loss and anesthesia-related vasodilation were compensated with colloid infusion as needed.

Routine anesthesia induction was performed using intravenous dexmedetomidine (1 ug/kg) or midazolam (0.05–0.075 mg/kg), cisatracurium (0.2 mg/kg), propofol (2–3 mg/kg), and sufentanil (0.1–0.3 μg/kg) to facilitate tracheal intubation. Anesthesia was maintained with propofol, sevoflurane, and a continuous infusion of remifentanil to achieve a bispectral index (BIS) value of 40–50 until completion of skin suturing. Cisatracurium (0.1 mg/kg) was administered hourly, with the final dose given at least 1 h before the end of surgery. Sufentanil (0.1 μg/kg) and flurbiprofen axetil (50 mg) were administered before discontinuation of remifentanil.

### Blinding

An anesthesiologist, adhering to the study protocol, administered anesthesia and was responsible for blood sampling and testing during the preoperative, intraoperative, and post-anesthesia care unit (PACU) periods. Although aware of the intraoperative ventilation strategy, this anesthesiologist did not participate in data collection or postoperative follow-up. Data collection was performed by full-time follow-up staff, and statistical analysis was conducted by a statistician who was blinded to the data collection process.

### Intraoperative respiratory parameter settings and adjustment

A volume-controlled ventilation strategy was used, with a tidal volume set to 8 mL/kg of ideal body weight (IBW) and an inspiratory-to-expiratory ratio of 1:2. IBW was calculated using the following formulas (12): 45.5 + 0.91× (centimeters of height - 152.4) for females and 50 + 0.91× (centimeters of height - 152.4) for males. Mixed air flow was restricted to 2–3 L/min, and the respiratory rate was adjusted to maintain EtCO_2_ between 35 and 45 mmHg. Positive end expiratory pressure (PEEP) was not applied, however, lung recruitment maneuvers (RMs) (13, 14) were employed to handle potential atelectasis immediately after tracheal intubation and every time when the ventilator was interrupted during the study.

### FiO_2_ settings

Immediately after tracheal intubation, FiO_2_ was set to 0.3. It was then incrementally increased by 0.1 steps until it reached 0.6, followed by a final increase to 0.8. Each FiO_2_ setting was maintained for at least 30 min before blood samples were drawn. Following this, FiO_2_ was adjusted as necessary to maintain PaO_2_ within the range of 100–200 mmHg. The final FiO_2_ value was recorded as dispayed on the anesthesia machine monitor.

### Blood gas analysis (BGA)

Blood samples were collected via the radial artery catheter into 1-mL heparinized syringes and immediately analyzed using a Siemens Rapidpoint 405 Co-oximeter (Siemens, Munich, Germany) at a satellite laboratory located in the operating room. The analyzer was calibrated automatically every 6 h. To ensure accuracy, blood samples were carefully deaerated, mixed, and analyzed promptly after collection. The primary outcome was PaO_2_, with additional data on the PaO_2_/FiO_2_ ratio, SpO_2_, hemoglobin (Hb), and other relevant parameters collected by an independent researcher.

### Sample size calculation

Based on previous similar studies (4, 15), we estimated that 50 patients would be sufficient for this prospective self-controlled pilot study. Although only 50 patients were enrolled, each participant underwent five interventions and outcomes, which was effectively equivalent to including 250 patients.

### Statistical analysis

Statistical analysis was conducted using R software (version 4.4.0). Normal variables were expressed as mean ± standard deviation (SD), while non-normally distributed variables were presented as median (interquartile range). Multivariable restricted cubic splines (RCS) were used to assess the linear relationship between continuous variables. Based on the characteristics of repeated measurement data, the repeated-measures correlation (rmcorr) analysis was first used to analyze the correlation between FiO_2_ and PaO_2_, calculated the repeated measures correlation coefficient (*r*), and evaluated the strength of the correlation between the two continuous variables based on the magnitude of the r value. To further quantified the impact of FiO_2_ on PaO_2_, linear mixed effects models were constructed. The model taken PO_2_ as the dependent variable, FiO_2_, as a fixed effect, the random intercept of patients and the random slope of FiO_2_ [formula: PO_2_, ~FiO_2_, + (1 + FiO_2_, ID)] were included in the random effects section to explain individual differences and heterogeneous responses among patients. To controled the influence of potential confounding factors, clinical related covariates such as age, gender, PCO_2_, PEEP, temperature, PH, HCT, and laparoscopic surgery or not were gradually introduced on the basis of the basic model to improve model accuracy and reduce residual variation. The model fitting adopted the Restricted Maximum Likelihood (REML) method. The convergence of the model was evaluated by gradient values, and the convergence criterion was set to a maximum gradient absolute value less than 0.002. The statistical significance was set to bilateral *a* = 0.05. Continuous variables were expressed as mean ± standard deviation or median (interquartile range), while categorical variables were expressed as frequency (percentage).

## Results

### Baseline clinical parameters

**Figure 1:**
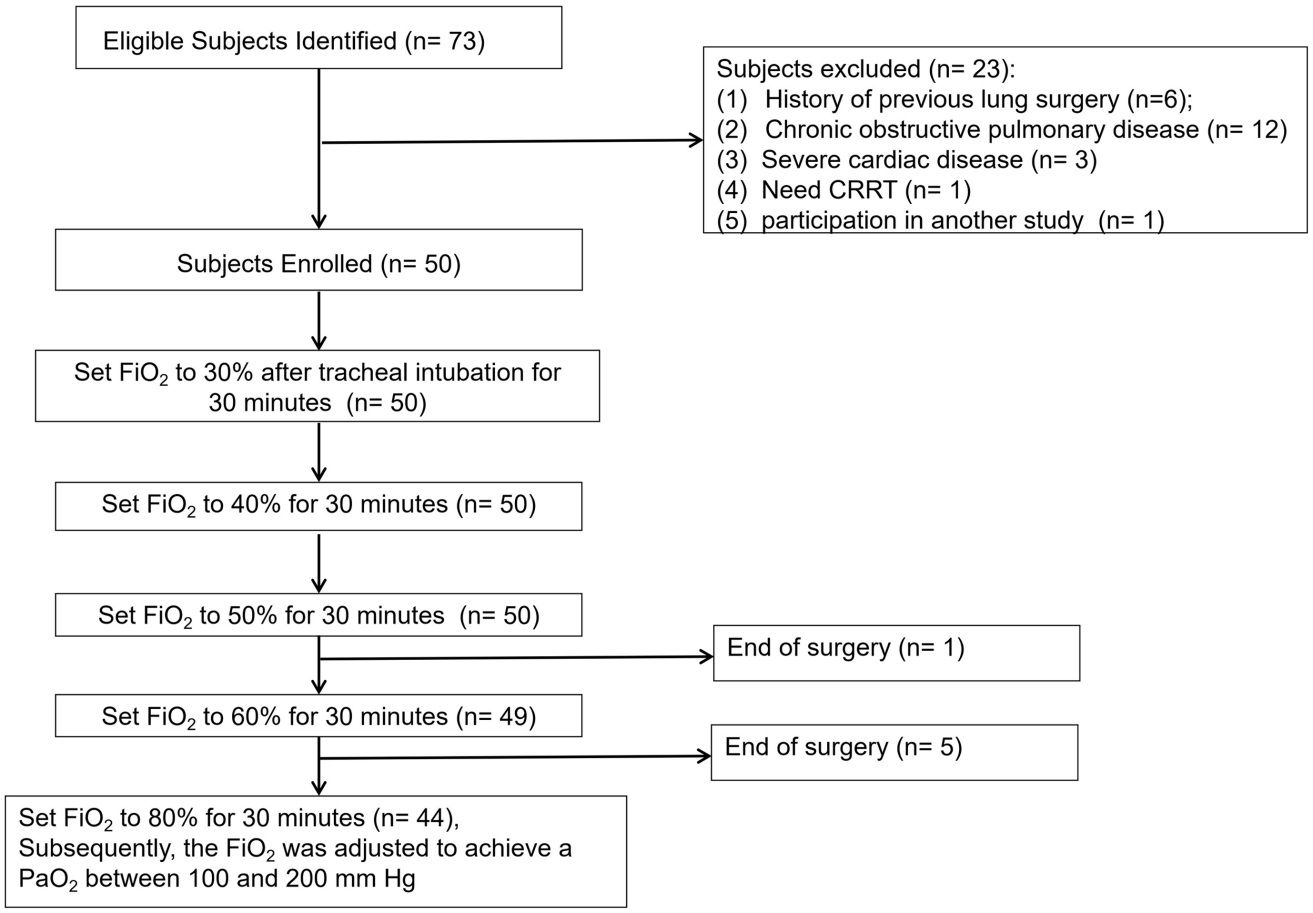
Consolidated standards of reporting trials statement flow diagram.

A total of 73 patients were initially recruited for the study, with the final analysis including 50 patients, as shown in Figure 1. The mean age of the participants was 62 years, and 56% were male. The majority of patients (70%) were classified as ASA physical status II. Additionally, 50% of patients underwent laparoscopic surgery. The duration of surgery and mechanical ventilation are detailed in Table 1.

**Table 1:** Baseline characteristics of patients undergoing general anesthesia (*n* = 50).

Preoperative characteristics	*n* = 50
Age (yr)	62 ± 12
Sex (male) [*n* (%)]	28 (56)
Weight (kg)	61 ± 11
Height (cm)	164 ± 8
ASA class (I/II/III) [*n*]	3/35/12
Coexistent disease [*n* (%)]	
Hypertension	20 (40)
Diabetes	3 (6)
Anaemia	1 (2)
Type of surgery, no. (%)	
Abdominal surgery	16 (32)
Neurosurgery	4 (8)
Gynaecology	2 (4)
Urology	7 (14)
Orthopaedics	21 (42)
Laparoscopic surgery, no. (%)	25 (50)
Robot-assisted laparoscopic surgery, no. (%)	7 (14)
Operative characteristics	
Duration of mechanical ventilation (min)	183 ± 52
Duration of surgery (min)	165 ± 46
Total I.V. fluid (ml)	1075 ± 249
Blood loss	310 ± 383

### Relationship between FiO_2_ and PaO_2_

**Figure 2:**
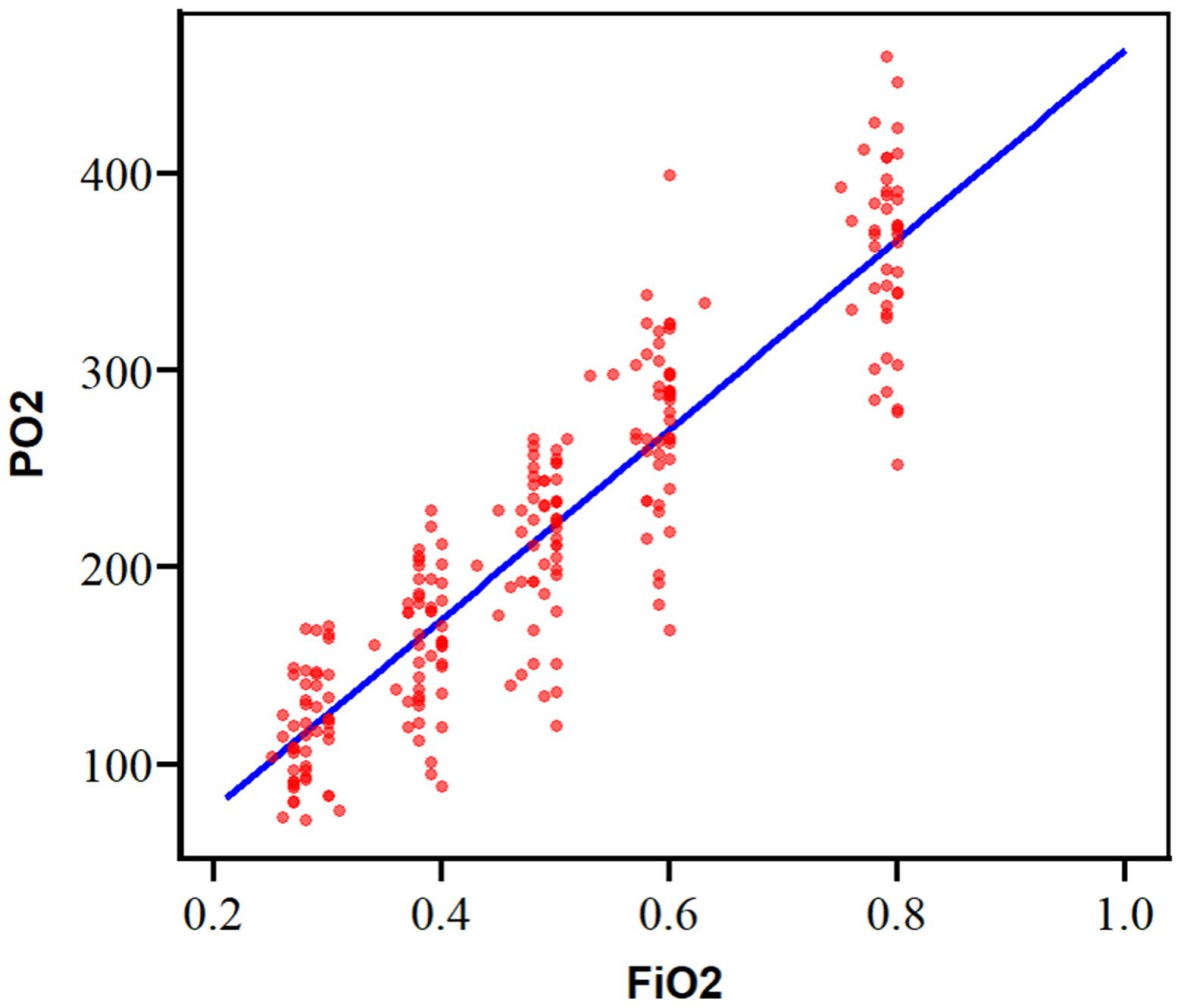
The rmcorr analyses of correlation between FiO_2_ and PaO_2_.

Rmcorr analyses revealed a significant correlation between FiO_2_ and PaO_2_ (*p* < 0.001) (Table 2). The correlation coefficient (*r*) was 0.967, indicating a predictive accuracy exceeding 80%. The relationship between FiO_2_ and PaO_2_ was described by the linear mixed-effects model: *Y* = −18.4 + 4.8*X (where *Y* = PaO_2_ and *X* = FiO_2_) (Figure 2 and Table 2). An FiO_2_ range of 25%–46% was found to maintain PaO_2_ within the 100–200 mmHg range. Model convergence was robust, with a gradient value of 0.00003. The fixed effects of model covariates were summarized in Supplementary Table S1.

**Table 2:** Rmcorr correlation coefficients and linear mixed-effects model parameters for FiO_2_ and PaO_2_.

	Total	Laparoscopic surgery	Non-laparoscopic surgery
Rmcorr analysis			
*r*	0.967	0.944	0.988
95% CI	0.956–0.975	0.926–0.958	0.985–0.991
*df*	191	189	193
*p*-value	<0.001	<0.001	<0.001
LMM: PO_2_ ~ FiO_2_ + (1 + FiO_2_|ID)			
*p*-value	<0.001	<0.001	<0.001
FiO_2_ slope	4.8097	4.384	5.218
Intercept	−18.449	−3.911	−32.406
Prediction equation	*Y* = −18.4 + 4.8*X	*Y* = −3.9 + 4.4*X	*Y* = −32.4 + 5.2*X
Gradient value	0.00003	0.00003	0.00003

*df*, degrees of freedom; CI, confidence interval.

**Figure 3:**
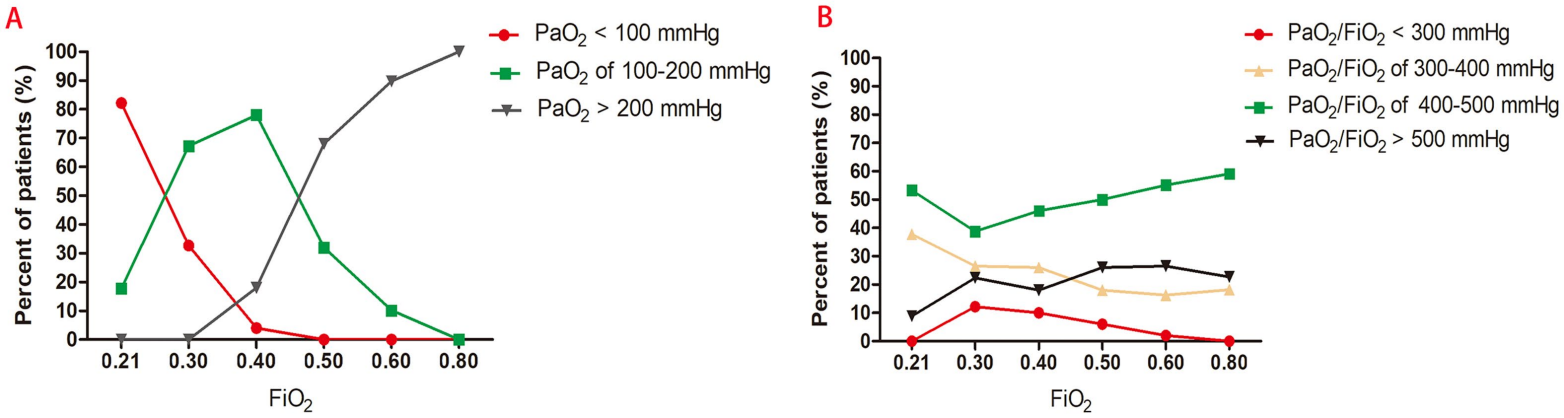
**(A)** Distribution of PaO_2_ under varying FiO_2_ levels; **(B)** Distribution of PaO_2_/FiO_2_ under varying FiO_2_ levels.

At an FiO_2_ of 40, 78.0% of patients maintained PaO_2_ between 100 and 200 mmHg. In contrast, 82.2% of patients had PaO_2_ values below 100 mmHg at 21% FiO_2_, while more than 68.0% exceeded 200 mmHg at 50% FiO_2_ levels (Table 3, Figure 3A).

**Table 3:** Distribution of patients by PaO_2_ and PaO_2_/FiO_2_ across FiO_2_ levels.

FiO_2_	PO_2_ (mm Hg), *n* (%)				PaO_2_/FiO_2_, *n* (%)		
	<100	100–200	>200	<300	300–400	400–500	>500
21%	37 (82.2%)	8 (17.8%)	0	0 (0%)	17 (37.8%)	24 (53.3%)	4 (8.9%)
30%	16 (32.7%)	33 (67.3%)	0	6 (12.2%)	13 (26.5%)	19 (38.8%)	11 (22.4%)
40%	2 (4.0%)	39 (78.0%)	9 (18.0%)	5 (10.0%)	13 (26.0%)	23 (46.0%)	9 (18.0%)
50%	0 (0%)	16 (32.0%)	34 (68.0%)	3 (6.0%)	9 (18.0%)	25 (50.0%)	13 (26.0%)
60%	0 (0%)	5 (10.2%)	44 (89.8%)	1 (2.0%)	8 (16.3%)	27 (55.1%)	13 (26.5%)
80%	0 (0%)	0 (0%)	44 (100%)	0 (0%)	8 (18.2%)	26 (59.1%)	10 (22.7%)

FiO_2_ = inspiratory oxygen fraction; PO_2_ = partial pressure of oxygen.

### Comparison of laparoscopic and non-laparoscopic surgery

In patients undergoing laparoscopic surgery, the linear mixed-effects model describing the relationship between FiO_2_ and PaO_2_ was: *Y* = −3.9 + 4.4*X*.

An FiO_2_ range of 24%–46% maintained PaO_2_ within 100–200 mmHg.

For non-laparoscopic surgery, the relationship was: *Y* = −32.4 + 5.2*X*.

An FiO_2_ range of 25%–45% achieved similar PaO_2_ control (Table 2). Both models demonstrated excellent convergence (gradient = 0.00003).

### Relationship between FiO_2_ and PaO_2_/FiO_2_

**Figure 4:**
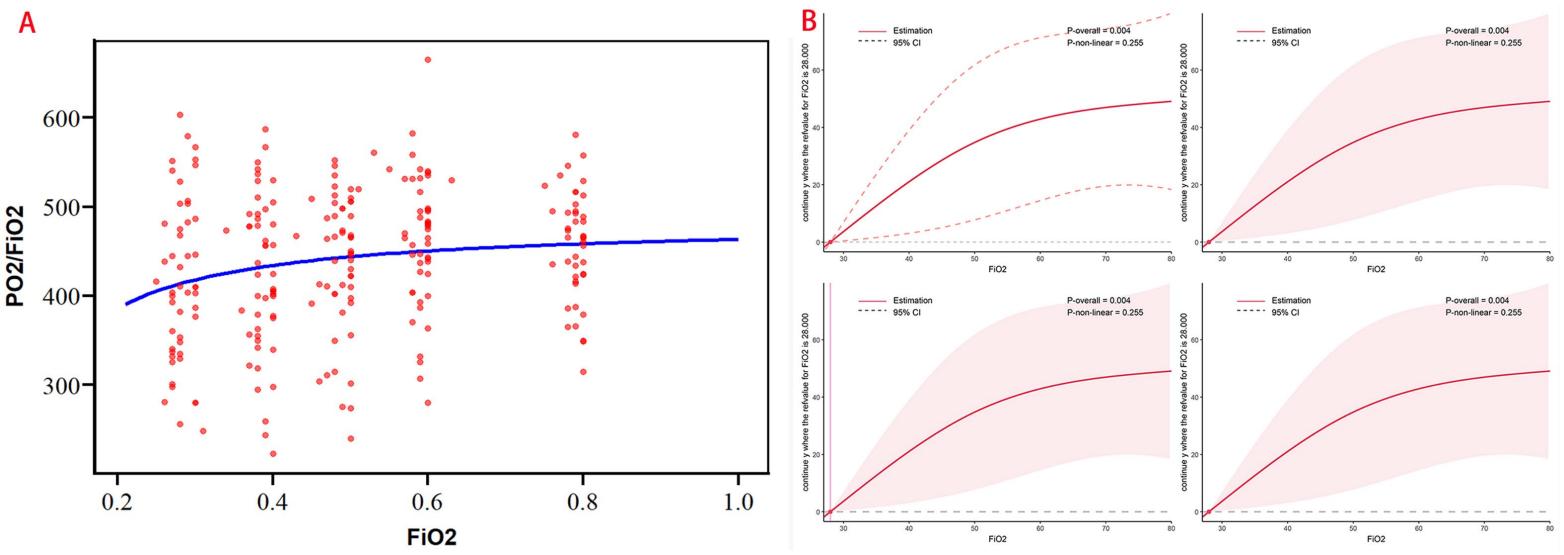
**(A)** The rmcorr analyses of correlation between FiO_2_ and PaO_2_/FiO_2_; **(B)** the RCS analysis of FiO_2_ and PaO_2_/FiO_2_.

Rmcorr analysis revealed a weak but statistically significant correlation between FiO_2_ and PaO_2_/FiO_2_ (*r* = 0.290; 95% CI: 0.155–0.414; *df* = 191; *p* < 0.001; Figure 4A). Adjusted restricted cubic spline (RCS) analysis showed no significant non-linear association between FiO_2_ and PaO_2_/FiO_2_ (*P* for non-linearity = 0.255; Figure 4B).

A PaO_2_/FiO_2_ ratio below 300 mmHg—indicative of pulmonary gas exchange dysfunction—was observed in 12.2% of patients at 30% FiO_2_, 6.0% at 50% FiO_2_, and only 2.0% at 60% FiO_2_. However, the proportion of patients with PaO_2_/FiO_2_ within the normal range (400–500 mmHg) did not significantly increase with rising FiO_2_ (*p* > 0.05), remaining between 46.0 and 59.1% across the 40%–80% FiO_2_ range. When FiO_2_ was ≥50%, more than 26.0% of patients exhibited PaO_2_/FiO_2_ values exceeding 500 mmHg (Table 3, Figure 3B).

### Blood gas analysis (BGA)

Detailed results from the blood gas analysis were presented in Supplementary Table S2. Significant differences in PaO_2_ were observed across the various FiO_2_ groups.

## Discussion

We observed a significant linear correlation between FiO_2_ and PaO_2_ during general anesthesia with dual-lung ventilation. We observed that at an FiO_2_ of 40, 78.0% of patients maintained PaO_2_ between 100 and 200 mmHg, while more than 68.0% exceeded 200 mmHg at 50% FiO_2_ levels. Our findings suggested that maintaining 40% FiO_2_ was generally sufficient for most patients to achieve PaO_2_ levels of 100–200 mmHg, unless specific clinical conditions require otherwise. Importantly, FiO_2_ need not exceed 50% in most patients undergoing general anesthesia with dual-lung ventilation.

The normal PaO_2_ for healthy adults typically ranged from 80 to 100 mmHg and served as a critical indicator for detecting tissue and organ hypoxia during surgery. According to the oxygen dissociation curve, when SpO_2_ reached 99%–100%, PaO_2_ was approximately 160 mmHg. The oxygenation index for normal human lungs was 400–500 (PaO_2_/FiO_2_), which implied that a theoretical FiO_2_ of 32%–40% would be needed to achieve a PaO_2_ of approximately 160 mmHg. Our findings further suggested that maintaining FiO_2_ between 25% and 46% was effective in sustaining PaO_2_ within the 100–200 mmHg range.

A multicenter cross-sectional study (11) highlighted that hyperoxemia and excessive oxygen exposure were prevalent during general anesthesia. The WHO and CDC have recently recommended an FiO_2_ of 80% during and immediately after surgery to reduce the risk of surgical site infections (SSIs) (16). However, concerns have been raised about the potential adverse effects of high FiO_2_, and some guidelines now recommend maintaining FiO_2_ ≤ 0.4 (17). Miller’s Anesthesiology (18) notes that anesthesia, whether delivered with spontaneous or mechanical ventilation, typically impaired lung function. A conventional practice has been to maintain FiO_2_ between 0.3 and 0.4 during mechanical ventilation, as higher concentrations might contribute to atelectasis.

Our study supported this approach, showing that maintaining FiO_2_ within a moderate range prevents excessive oxygen exposure and optimizes oxygenation. Our analysis revealed a significant linear correlation between FiO_2_ and PaO_2_, with *R* values of 0.967 in rmcorr analyses. Several theoretical models (5–8) have attempted to predict the relationship between FiO_2_ and PaO_2_, but most of these models were designed for ICU patients receiving various ventilatory modes (e.g., volume-controlled, pressure-controlled ventilation). These models might not be directly applicable to intraoperative settings, where different ventilation strategies could influence ventilation/perfusion matching, cardiac output, and venous oxygen saturation (SvO_2_), which could impact the accuracy of the predictions. Additionally, models such as those by Al-Otaibi and Hardman focus on narrower FiO_2_ ranges (40%–60%) (9, 10), and no studies have systematically explored a conservative approach to intraoperative oxygen therapy. Our clinical findings provided valuable data for this context, confirming the linear relationship between FiO_2_ and PaO_2_ during general anesthesia. We also noted despite changes in FiO_2_, SpO_2_ remained high and clustered near saturation, highlighting the limitation of SpO_2_ monitoring but also suggesting that reliance on SpO_2_ alone could mask hyperoxemia.

In this study, we observed the weak correlation between FiO_2_ and PaO_2_/FiO_2_ in both repeated-measures and restricted cubic spline analyses. First, the PaO_2_/FiO_2_ ratio remained more informative for evaluating pulmonary gas exchange, particularly in patients with ventilation–perfusion (V/Q) mismatch or diffusion impairment. In such cases, PaO_2_ might not rise in proportion to FiO_2_, indicating diffusion limitation or alveolar shunting rather than insufficient oxygen delivery. Secondly, the absence of a linear increase in PaO_2_/FiO_2_ at higher FiO_2_ levels likely reflected physiological saturation effects, including the plateau of the oxygen–hemoglobin dissociation curve. Similar findings have been reported in both perioperative and critical care studies, which demonstrate that increasing FiO_2_ often fails to improve PaO_2_ proportionally and may even exacerbate V/Q mismatch (19, 20).

Finally, prior research has shown that a period of 5–10 min was typically sufficient for PaO_2_ to equilibrate after a change in FiO_2_ in ICU patients (21). In this study, each FiO_2_ level was maintained for at least 30 min, providing ample time for PaO_2_ to stabilize following changes in FiO_2_.

A recent large observational cohort study involving 350,647 patients demonstrated that exposure to supraphysiological oxygen concentrations during surgery was associated with an increased incidence of kidney, myocardial, and pulmonary injury (22). Numerous studies have also linked high intraoperative FiO_2_ with increased risk of both cardiac (23) and pulmonary complications (24), while others have found no such association (16, 25). Even routine preoxygenation with 100% oxygen during anesthesia induction could lead to atelectasis (26), prompting recommendations to decrease FiO_2_ after induction to mitigate further atelectasis formation (27). High perioperative FiO_2_ might mask deteriorating oxygenation if it exceeded the levels required to maintain adequate SpO_2_ (28). Several retrospective studies have also suggested a dose-dependent relationship between intraoperative FiO_2_ and postoperative respiratory complications or 30-day mortality (1, 29). However, the present study did not investigate the impact of FiO_2_ on clinical outcomes, as this was beyond the scope of the current investigation.

The study focuses on the FiO_2_–PaO_2_ correlation but did not evaluate patient-centered outcomes (e.g., postoperative atelectasis, SSI, or respiratory mortality), thus providing limited guidance for clinical oxygen therapy decisions. However, recent randomized clinical trial (2) and retrospective study (1) have highlighted the association between elevated intraoperative FiO_2_ and an increased risk of major respiratory complications and mortality, often in a dose-dependent manner. Thus this prospective pilot study aimed to explore physiological correlation between FiO_2_ and PaO_2_ in patients undergoing general anesthesia with dual lung ventilation by volume-controlled ventilation strategy.

Clinically, these results highlighted the need for individualized oxygen therapy rather than the routine administration of high FiO_2_ during anesthesia and mechanical ventilation. Targeting a PaO_2_ between 100 and 200 mmHg might provide adequate oxygenation while minimizing hyperoxia-related injury. Future research should aim to develop real-time, physiology-based FiO_2_ titration strategies that integrate continuous gas exchange monitoring, dynamic compliance, and shunt estimation to optimize oxygen delivery during the perioperative period.

### Limitations

This study has several limitations. First, it was conducted at a single center and included a relatively specific patient population (ASA physical status I–III, elective surgery, and dual-lung ventilation), which limits the generalizability of the findings to broader clinical settings, including emergency surgery or critically ill patients. Moreover, because patients with pulmonary disease were excluded, the results cannot be extrapolated to individuals with compromised lung function, who are often at increased risk of oxygenation abnormalities. Future studies should include such populations to determine whether the relationship between inspired oxygen fraction (FiO_2_) and arterial oxygen tension (PaO_2_) differs in patients with impaired pulmonary function. Notably, arterial blood gas (ABG) analysis is routinely performed in critically ill patients or those with pulmonary disease, which may facilitate more precise titration of FiO_2_ to achieve normoxemia.

Second, although adjustments were made for body temperature, hemoglobin concentration, and pH, other important physiological variables—such as cardiac output, lung compliance, and ventilation–perfusion mismatch—were not assessed and may have influenced PaO_2_ measurements. Nevertheless, this was a self-controlled study, and each patient served as their own control under different FiO_2_ conditions, thereby minimizing interindividual variability. Consequently, baseline clinical characteristics, including smoking history, dynamic lung compliance, intrapulmonary shunt fraction, and the intraoperative anesthesia settings, were remained consistent within each FiO_2_ conditions.

Thirdly, the results might differ with other ventilatory modes (pressure-controlled, spontaneous breathing), however, a volume-controlled ventilation strategy was the most commonly used mode. Fourth, in this study the FiO_2_ was altered stepwise in steps from 0.3 to 0.8. Therefore, the relationship between FiO_2_ and PaO_2_ could not be evaluated beyond this range. Finally, as a single-center pilot study with 50 patients, the external validity of the results may be limited. Despite enrolling only 50 participants, each underwent six blood gas evaluations, yielding a total of 300 data points, which provided adequate statistical power for constructing a reliable model. Multi-center studies with larger cohorts were needed to confirm the FiO_2_–PaO_2_ relationship across diverse patient populations and clinical settings, and add postoperative respiratory complications, duration of mechanical ventilation, or markers of lung injury to connect physiological findings with clinical relevance.

## Conclusion

A significant linear correlation was identified between FiO_2_ and PaO_2_ during general anesthesia with dual-lung ventilation. In this prospective pilot study, our findings suggested that maintaining 40% FiO_2_ was generally sufficient for most patients to achieve PaO_2_ levels of 100–200 mmHg, unless specific clinical conditions require otherwise. Importantly, FiO_2_ need not exceed 50% in most patients undergoing general anesthesia with dual-lung ventilation.

## Data Availability

The raw data supporting the conclusions of this article will be made available by the authors, without undue reservation.
