# Supplementary material for: FiO2 requirements during general anesthesia with dual-lung ventilation: a prospective pilot study
Source: Front Med (Lausanne). 2026 Mar 9;13:1780890. doi: 10.3389/fmed.2026.1780890 (PMC13006499; doi:10.3389/fmed.2026.1780890)
Supplement: Supplementary file 1 [file Table_1.doc]

| **Supplementary Table 1. Relationship between factors and PaO2 by Rmcorr analysis and the linear mixed effects models.** | | | | | | | |
| --- | --- | --- | --- | --- | --- | --- | --- |
|  | Rmcorr | Linear mixed effects models | | | | |  |
| Factors | r | Estimate | Std. Error | df | t-value | P-value | VIF |
| FiO_2_ (%) | 0.9665 | 4.7277 | 0.1633 | 55.7577 | 28.953 | <0.001 | 1.19 |
| PCO_2_ (mmHg) | 0.0944 | -0.4456 | 0.6162 | 188.0715 | -0.723 | 0.4704 | 2.39 |
| PEEP (cmH_2_0) | 0.0004 | -1.7884 | 1.2651 | 167.1461 | -1.414 | 0.1593 | 1.07 |
| Temperature (℃) | -0.4685 | -7.2991 | 6.5158 | 160.5448 | -1.12 | 0.2643 | 1.18 |
| PH | -0.3124 | 28.2208 | 73.585 | 197.5143 | 0.384 | 0.7018 | 2.55 |
| HCT (%) | -0.5565 | -0.4763 | 0.5775 | 99.3112 | -0.825 | 0.4115 | 1.17 |
| Age (Year) | NA | -0.7834 | 0.3192 | 37.775 | -2.454 | 0.0188 | 1.12 |
| Gender | NA | 15.3918 | 8.3839 | 43.305 | 1.836 | 0.0732 | 1.24 |
| Laparoscopic surgery or not | NA | -0.9565 | 8.2493 | 44.9175 | -0.116 | 0.9082 | 1.19 |
| Note: Repeated measures correlation (rmcorr) is indicated by NA for static or categorical variables, for which the method is unsuitable. Furthermore, the convergence of the linear mixed-effects models was robust, with a minimal gradient value of 0.00003. Additionally, despite the known physiological influence of PCO_2_ on pH, which can cause multicollinearity, a variance inflation factor (VIF) analysis confirmed that all values were below the threshold of 5, indicating that multicollinearity does not substantially affect the stability of the model estimates. | | | | | | | |
