# Supplementary material for: FiO2 requirements during general anesthesia with dual-lung ventilation: a prospective pilot study
Source: Front Med (Lausanne). 2026 Mar 9;13:1780890. doi: 10.3389/fmed.2026.1780890 (PMC13006499; doi:10.3389/fmed.2026.1780890)
Supplement: Supplementary file 2 [file Table_2.doc]

| **Supplementary Table 2. Blood Gas Analysis (BGA) results.** | | | | | | | |
| --- | --- | --- | --- | --- | --- | --- | --- |
|  |  | FIO_2_ =21% | FIO_2_ =30% | FIO_2_ =40% | FIO_2_ =50% | FIO_2_ =60% | FIO_2_ =80% |
| SpO_2_ (%) |  | 97.1±1.2 | 97.4±1.6 | 97.1±1.2 | 98.8±1.2 | 99.4±0.7 | 99.6±0.6 |
| T (°C) |  | 36.5±0.3 | 36.3±0.4 | 36.1±0.4 | 36.0±0.5 | 36.0±0.5 | 36.0±0.5 |
| PH |  | 7.42±0.02 | 7.38±0.04 | 7.35±0.05 | 7.34±0.05 | 7.35±0.05 | 7.35±0.05 |
| Hb (g/L) |  | 123±18 | 113±19 | 110±19 | 107±18 | 104±19 | 101±18 |
| Hct (%) |  | 36±10 | 35±6 | 34±6 | 33±5 | 32±6 | 31±5 |
| PO_2_ (mm Hg) |  | 88±13 | 118±28 | 164±34 | 215±39 | 272±48 | 358±51 |
| PaO_2_/FIO_2_ |  | 421±61 | 416±92 | 424±86 | 441±77 | 461±77 | 457±60 |
| PCO_2_ (mm Hg) |  | 37±3 | 39±5 | 40±6 | 41±6 | 40±6 | 40±5 |
| O_2_Ct (Vol%) |  | 16.6±2.5 | 15.3±2.5 | 15.3±2.6 | 15.1±2.5 | 14.9±2.7 | 14.7±2.4 |
| Total CO_2_ (mmol/L Hg) |  | 24.3±2.0 | 23.2±1.7 | 22.8±1.8 | 22.7±1.7 | 22.5±2.0 | 22.5±1.9 |
| HCO_3_ (mmol/L Hg) |  | 23.5±1.9 | 22.1±1.7 | 21.5±1.7 | 21.5±1.6 | 21.3±1.9 | 21.3±1.8 |
| FiO_2_ =inspiratory oxygen fraction; PaO_2_ =partial pressure of oxygen; O_2_Ct =oxygen content. | | | | | | | |
